# Low Risk of Resistance Evolution of *Spodoptera frugiperda* to Lufenuron and Chlorfenapyr in China

**DOI:** 10.3390/insects17100994

**Published:** 2026-09-25

**Authors:** Yunchao Wang, Wenling Ma, Bo Pang, Zhongjiu Xiao, Peizhi Tang, Ying Meng, Liangting Zhu, Jiawen Guo, Kun Yang

**Affiliations:** 1 School of Ecology, Zunyi Normal University, Zunyi 563006, China; wyc801suo@163.com (Y.W.); xzj198099@163.com (Z.X.); 2 Key Laboratory of Agri-Products Quality and Biosafety (Ministry of Education), Anhui Province Key Laboratory of Crop Integrated Pest Management, School of Plant Protection, Anhui Agricultural University, Hefei 230036, China; mwl@ahau.edu.cn; 3 College of Agriculture and Animal Husbandry of Xizang Autonomous Region, Lhasa 850000, China; 4 Shandong Engineering Research Center for Environment-Friendly Agricultural Pest Management, College of Plant Health and Medicine, Qingdao Agricultural University, Qingdao 266109, China; 5 Academy of Dongying Efficient Agricultural Technology and Industry on Saline and Alkaline Land in Collaboration with Qingdao Agricultural University, Dongying 257091, China; 6 Nanjing Hanguang Testing Technology Co., Ltd., Nanjing 210046, China; 7 State Key Laboratory for Quality and Safety of Agro-Products, Institute of Plant Protection and Microbiology, Zhejiang Academy of Agricultural Sciences, Hangzhou 310021, China; 8 Key Laboratory of Integrated Pest Management on Crops in East China, Ministry of Agriculture and Rural Affairs, Nanjing Agricultural University, No. 1 Weigang, Nanjing 210095, China

**Keywords:** *Spodoptera frugiperda*, chemical pesticide, insecticide resistance, fitness cost

## Abstract

*Spodoptera frugiperda* is a highly destructive migratory pest that severely restricts global agricultural production. Chemical insecticides remain the primary means of field population control, yet the resistance evolution risk of *S. frugiperda* to two commonly applied insecticides, lufenuron and chlorfenapyr, remains poorly clarified in China. In this study, we found that the four field *S. frugiperda* populations collected from Jiangsu, Guangxi, Guizhou, and Yunnan Provinces exhibited susceptible or low-level resistance to chlorfenapyr, lufenuron, emamectin benzoate, and spinetoram. However, moderate resistance to chlorantraniliprole (resistance ratio > 5) was detected in the Guangxi and Guizhou populations, consistent with regional variation in diamide insecticide pressure. After 10 consecutive generations of indoor resistance screening, the selected resistant strains showed only 2.89-fold and 3.40-fold resistance increases to lufenuron and chlorfenapyr, respectively, indicating an extremely slow resistance development rate. Furthermore, the lufenuron-resistant and chlorfenapyr-resistant strains displayed negligible cross-resistance to emamectin benzoate, chlorantraniliprole, and spinetoram. Significant fitness costs were detected in both resistant strains, with marked declines in multiple growth, development and reproductive indicators. Collectively, these findings confirm that Chinese field *S. frugiperda* populations have a low evolutionary risk of resistance to lufenuron and chlorfenapyr, and resistance emergence is constrained by obvious fitness trade-offs. This study provides empirical evidence and theoretical guidance for the scientific formulation of *S. frugiperda* chemical control and resistance management strategies in China, while highlighting the need for region-specific monitoring of diamide resistance.

## Simple Summary

*Spodoptera frugiperda* is a destructive pest that threatens food crops such as corn worldwide. Farmers commonly rely on chemical insecticides to control its population, yet pests can evolve resistance over time, diminishing the effectiveness of these chemicals. In this study, we assessed the risk of *S. frugiperda* populations in China developing resistance to two widely used insecticides—lufenuron and chlorfenapyr. Larvae collected from four provinces remained largely susceptible to both compounds. We then exposed successive generations of *S. frugiperda* to these insecticides under laboratory conditions. Even after ten generations of continuous exposure, resistance developed only slowly and remained at a low level. Importantly, these resistant larvae showed no cross-resistance to other classes of insecticides, and they exhibited clear fitness costs, with reduced survival, growth, and reproduction compared with non-resistant larvae. Our findings indicate that these two insecticides carry a low risk of resistance development in *S. frugiperda*.

## 1. Introduction

*Spodoptera frugiperda* (J. E. Smith, 1797) (Lepidoptera: Noctuidae) is a typical polyphagous migratory insect pest with a host range covering more than 350 plant species, including major food and cash crops such as maize, rice, and cotton [1,2,3,4,5]. Originating from the Americas, this pest has rapidly spread worldwide, invading numerous countries across North America, South America, Africa, and Asia, causing severe crop yield losses and threatening food security [6,7,8,9]. A recent global review estimated that *S. frugiperda* can cause up to 70% yield losses in affected regions and annual economic losses exceeding US$13 billion, further highlighting its threat to global food security [5]. In China, *S. frugiperda* was first discovered in Yunnan Province in 2019, and has since spread to at least 13 provincial administrative regions, becoming a major persistent pest restricting national maize production [10,11].

Chemical insecticides are the core and most efficient tool for large-scale emergency prevention and control of *S. frugiperda*, playing an irreplaceable role in suppressing field pest populations [12,13,14]. However, long-term and unreasonable pesticide application has induced a series of ecological and agricultural problems, among which insecticide resistance evolution is the most prominent [15]. The mechanisms underlying insecticide resistance in *S. frugiperda* generally involve target-site mutations and metabolic detoxification mediated by cytochrome P450 monooxygenases, glutathione S-transferases, and esterases [5]. Pest resistance not only reduces the control efficacy of conventional insecticides and increases pesticide application dosage and frequency but also easily triggers pest resurgence, secondary pest outbreaks, and pesticide residue pollution [16,17]. Moreover, ongoing climate change further expands the overwintering range of *S. frugiperda* and accelerates the evolution of pesticide resistance, exacerbating the difficulty of field prevention and control [18]. At present, domestic research on the resistance characteristics and evolutionary rules of *S. frugiperda* to commonly used insecticides are still insufficient, which restricts the optimization of targeted resistance management strategies.

Given the escalating resistance of Chinese *S. frugiperda* populations to commonly used insecticides such as organophosphates, pyrethroids, and diamides [19,20], there is an urgent need for alternative compounds with novel modes of action. Lufenuron and chlorfenapyr, which target chitin synthesis and mitochondrial oxidative phosphorylation, respectively, possess mechanisms entirely distinct from existing resistance genotypes and are theoretically unlikely to confer cross-resistance. Notably, chitin synthase mutations associated with lufenuron resistance have already been detected in Chinese *S. frugiperda* [21], indicating that resistance alleles to this insecticide are present in field populations. Although resistance to these active ingredients has been reported internationally in a few other pests–including chlorfenapyr resistance in *Tetranychus urticae* from Australia [19] and *Helicoverpa armigera* [20]—their resistance evolution risk, cross-resistance spectrum, and associated fitness costs in Chinese *S. frugiperda* remain largely uncharacterized [21], their resistance evolution risk, cross-resistance spectrum, and associated fitness costs in Chinese *S. frugiperda* remain uncharacterised. Therefore, this study was designed to address this knowledge gap by systematically evaluate these parameters in Chinese populations of *S. frugiperda*, thereby providing a scientific basis for incorporating these two insecticides into sustainable resistance management rotation programmes.

Lufenuron and chlorfenapyr are two high-efficiency, broad-spectrum insecticides widely used for *S. frugiperda* control in Chinese farmlands, with unique insecticidal mechanisms and good field application prospects. Lufenuron is a classic benzoylurea insect growth regulator, which inhibits insect chitin synthesis, interferes with normal molting and growth and development of pests, and has the characteristics of high efficiency and low toxicity to non-target organisms [22,23]. Existing studies have clarified the resistance mechanism of multiple insects to lufenuron: mutation of the chitin synthase A (*CHSA*) gene in *S. frugiperda* can significantly improve lufenuron resistance [23], and overexpression of the cytochrome P450 gene *CYP12a4* also mediates lufenuron resistance in *Drosophila melanogaster* Meigen, 1830 (Diptera: Drosophilidae) [24].

Chlorfenapyr, a pyrazole insecticide and uncoupler of mitochondrial oxidative phosphorylation, exerts its insecticidal effect by disrupting energy metabolism in pests. In the absence of defined resistance target proteins or associated functional genes, this mode of action is theoretically associated with a low resistance-induction potential [24,25]. Nevertheless, previous studies have confirmed that multiple pest species can develop low-to-medium level resistance to chlorfenapyr after long-term selection, such as 2.9-fold resistance in Australian two-spotted mites, 74-fold resistance in Pakistani bollworms, and 7.7-fold resistance in *Earias vittella* [19,20]. Despite the above research foundations, the field resistance level, resistance development rate, cross-resistance spectrum and fitness cost characteristics of Chinese *S. frugiperda* populations to lufenuron and chlorfenapyr remain unclear, which urgently needs systematic verification and evaluation.

Thus, this study aimed to systematically evaluate the resistance evolution risk of Chinese populations of *S. frugiperda* to lufenuron and chlorfenapyr, and to provide scientific support for rational pesticide rotation and sustainable pest management.

## 2. Materials and Methods

### 2.1. Collection and Rearing of Spodoptera frugiperda

Two categories of *S. frugiperda* strains were utilized in this study: a laboratory susceptible reference strain and four field-collected populations. The susceptible strain was originally collected from maize fields in Jurong, Nanjing, Jiangsu Province (119.16° E, 31.96° N), and subsequently reared under insecticide-free laboratory conditions for more than 10 generations to establish a stable, genetically susceptible population. This strain served as the reference baseline for resistance ratio calculations in both field populations and laboratory-selected strains, and also as the parental starting population (F_0_) for the subsequent 10-generation resistance selection experiments with lufenuron and chlorfenapyr. The four field populations were collected from typical maize-producing areas in Huaian of Jiangsu (119.02° E, 33.60° N), Nanning of Guangxi (108.32° E, 22.82° N), Guiyang of Guizhou (106.27° E, 26.11° N), and Kunming of Yunnan (102.71° E, 25.04° N) (Figure 1) and were used to assess the current field resistance status against five commonly used insecticides. The Jiangsu field population was collected separately from a different set of maize fields in the same province to ensure independence from the laboratory susceptible strain. All strains were reared under identical conditions at 25 ± 1 °C, 60–70% relative humidity, and a 16 h light: 8 h dark photoperiod, with fresh corn leaves provided as daily feed. Larvae from field populations were reared to the third instar prior to toxicity bioassays, while the susceptible strain was maintained continuously for both baseline determination and resistance selection.

### 2.2. Pesticides

All insecticides used in the bioassays were commercially available high-purity technical reagents: 96% lufenuron (Zhejiang Shijia Technology Co., Ltd., Huzhou, China), 98% chlorfenapyr (Shandong Hailier Chemical Co., Ltd., Qingdao, China), 98% emamectin benzoate (Nanjing Red Sun Co., Ltd., Nanjing, China), 98% chlorantraniliprole (Shandong Weifang Rainbow Chemical Co., Ltd., Weifang, China), and 81.2% spinetoram (Corteva Agriscience, Shanghai, China).

### 2.3. Toxicity Bioassay

The toxicity bioassay was performed based on the improved artificial diet feeding method [7]. Each insecticide was diluted with acetone to prepare 6 gradient concentrations. The solidified artificial diet was divided into 20 g equal portions and placed in sealed ziplock bags. Then 1 mL of each gradient pesticide solution was fully mixed with the artificial diet to prepare treated feed, and blank acetone treatment was set as the control group. All treated diets were prepared freshly on the day of bioassay and used immediately to minimize potential degradation or volatilization of the test compounds. Each concentration was thoroughly homogenized with the diet to ensure uniform distribution of the insecticide, and the nominal concentrations were calculated based on the dilution series. The treated artificial diets were placed in ventilated Petri dishes, and 15 healthy and uniformly growing 3-instar *S. frugiperda* larvae were inoculated into each dish. Each treatment was set up with three biological replicates. After 48 h, the larval mortality of each group was counted. Larvae were judged as dead if no active movement was observed after gentle brushing with a writing brush, or obvious poisoning symptoms such as body malformation, convulsion, and inability to feed appeared.

### 2.4. Fitness and Life-Table Indicators of Resistant and Susceptible S. frugiperda Strains

To evaluate the fitness differences among susceptible strain, lufenuron-resistant strain and chlorfenapyr-resistant strain, paired adult insects emerging on the same day were selected and placed in glass test tubes (2.5 cm × 8 cm) lined with weighing paper. Thirty pairs of adults were set for each strain, with three replicates. The test tubes were sealed with degreased cotton soaked in honey water to provide adult nutrition, and fresh corn leaves were placed at the bottom of the tubes and replaced daily. The rearing environment was consistent with the standard conditions (25 ± 1 °C, 60–70% relative humidity, 16:8 h light-dark cycle). The daily egg laying amount of female adults and adult lifespan were recorded until all adults died. Eggs laid on the same day were collected and transferred to insect rearing boxes for hatching observation, with 30 replicates for each strain. The egg hatching time and hatching rate were recorded daily. Subsequently, 100 newly hatched larvae of each strain were selected for unified rearing, with 30 replicates. Larval feed was replaced daily, and key developmental indicators including larval development duration, pupation rate and pupal weight were continuously observed and recorded. Sixty uniformly developed pupae of each strain were selected and reared in single independent rearing boxes, with three replicates. The adult emergence status was observed daily, and the emergence rate and population sex ratio were statistically recorded. Based on the recorded life-table parameters, the net reproductive rate (R_0_) and the relative fitness of resistant strains were calculated. The net reproductive rate was calculated by the formula R_0_ = N_t+1_/N_t_, where N_t_ represents the initial population quantity and N_t+1_ represents the population quantity of the next generation after reproduction. The relative fitness of resistant strains was defined as the ratio of their net reproductive rate to that of the susceptible strain.

### 2.5. Data Analysis

All experiments in this study were conducted with three biological replicates, each containing multiple individuals (15 larvae per concentration for toxicity bioassays, 30 adult pairs for fecundity assays, and 300 larvae for life-table observations). The use of three replicates provided sufficient statistical power, as confirmed by the small standard errors obtained for all measured parameters. PoloPlus software, version 2.0 (LeOra Software, Berkeley, CA, USA) was used to calculate the median lethal concentration (LC_50_), 95% confidence limits (CL), and regression slope of each insecticide against different *S. frugiperda* strains. Cross-resistance was evaluated based on the resistance ratio (RR), calculated as LC_50_ of the resistant strain divided by LC_50_ of the susceptible strain. Following the criteria recommended by the World Health Organization, RR values < 5 were classified as susceptible or no cross-resistance, RR values between 5 and 10 as moderate resistance, and RR values > 10 as high resistance [26]. Significant differences in LC_50_ values between groups were determined by the non-overlapping of 95% CL. Probit analysis was used to estimate the theoretical mortality and chi-square value (*χ*^2^) of each bioassay. Prior to ANOVA, all data were tested for normality using the Shapiro–Wilk test and for homogeneity of variance using Levene’s test. All data met the assumptions of normality and homogeneity of variance; therefore, no transformation was required. The response variables were corrected mortality for the three-way ANOVA, and life-table parameters (egg duration, egg hatchability, larval duration, larval survival rate, pupation rate, pupal weight, pupal period, adult emergence rate, sex ratio, adult longevity, fecundity, net reproductive rate, and relative fitness) for the one-way ANOVA. A three-way ANOVA was used to analyze the effects of geographical region, insecticide type, and concentration on corrected mortality. One-Way ANOVA followed by Tukey’s honestly significant difference (Tukey’s HSD) post-hoc test was used to compare the differences in life-table and fitness indicators between susceptible and resistant strains. All statistical analyses were performed using SPSS 21.0 software.

## 3. Results

### 3.1. Relative Toxicity of Insecticides to Different Geographical S. frugiperda Populations

A three-way ANOVA was performed to evaluate the effects of geographical region, insecticide type, and concentration on the corrected mortality of *S. frugiperda* (Table 1). The analysis revealed significant main effects of region and concentration, but not of insecticide. Significant two-way interactions were detected between region and insecticide, as well as between insecticide and concentration, indicating that the toxicity of the tested insecticides varied across regions and concentrations, whereas the three-way interaction among region, insecticide, and concentration was not significant.

The toxicity determination results of five insecticides against four geographical *S. frugiperda* populations are shown in Table 2. For lufenuron, the Jiangsu (JS) population had the highest sensitivity with a LC_50_ value of 51.96 mg/L, followed by Yunnan (YN, 57.97 mg/L) and Guizhou (GZ, 92.99 mg/L) populations. The Guangxi (GX) population showed the lowest sensitivity, with a LC_50_ of 129.98 mg/L. Compared with the indoor susceptible strain, the resistance ratios of JS, GX, GZ and YN populations to lufenuron were 1.13, 2.84, 2.03 and 1.26, respectively, all lower than 5, indicating that all field populations were either susceptible or showed low-level insensitivity to lufenuron. For chlorfenapyr, the GZ population had the highest sensitivity (LC_50_ = 6.17 mg/L), followed by YN (6.22 mg/L) and JS (11.84 mg/L) populations, while the GX population had the lowest sensitivity (21.48 mg/L). The corresponding resistance ratios were 1.06 (GZ), 1.07 (YN), 2.03 (JS) and 3.68 (GX), all below 5, confirming that all field populations remained susceptible to chlorfenapyr with no obvious resistance development. Emamectin benzoate showed the highest toxicity to the YN population (LC_50_ = 3.43 mg/L), followed by GZ (5.43 mg/L), JS (5.78 mg/L) and GX (8.32 mg/L) populations, with resistance ratios ranging from 0.99 to 2.40. Chlorantraniliprole exhibited high toxicity to YN (1.54 mg/L) and JS (1.65 mg/L) populations, while the toxicity to GZ (11.54 mg/L) and GX (18.18 mg/L) populations decreased significantly, with resistance ratios of 0.71, 0.76, 5.34 and 8.42 for YN, JS, GZ and GX populations, respectively; notably, GX and GZ populations developed medium-level resistance (Resistance ratio > 5) to chlorantraniliprole. Spinetoram had the highest toxicity to the YN population (0.19 mg/L), followed by JS (0.28 mg/L), GZ (0.43 mg/L) and GX (0.76 mg/L) populations, with resistance ratios between 0.90 and 3.62, presenting susceptible or low resistance levels.

### 3.2. Developmental Patterns of Indoor Resistance to Lufenuron and Chlorfenapyr

Continuous indoor resistance selection was performed on *S. frugiperda* susceptible strain with lufenuron and chlorfenapyr via artificial diet mixing method, and the dynamic changes in resistance ratios across 10 consecutive generations were statistically analyzed (Table 3). The results showed that the resistance of *S. frugiperda* larvae to both insecticides increased slowly and steadily with the increase in selection generations, without rapid resistance development. After 10 generations of continuous selection, the LC_50_ value of lufenuron-resistant strain increased from 45.83 mg/L (F_0_ generation) to 132.47 mg/L (F_10_ generation), with a final resistance ratio of 2.89. For chlorfenapyr, the LC_50_ value rose from 5.84 mg/L (F_0_ generation) to 19.84 mg/L (F_10_ generation), and the final resistance ratio was 3.40. Throughout the selection process, the resistance growth rate of both strains was stable and slow, indicating that *S. frugiperda* has an extremely low potential to develop high-level resistance to lufenuron and chlorfenapyr under continuous single pesticide pressure.

### 3.3. Cross-Resistance of Lufenuron- and Chlorfenapyr-Resistant S. frugiperda Strains to Other Insecticides

The cross-resistance levels of F_10_ generation lufenuron-resistant and chlorfenapyr-resistant strains to emamectin benzoate, chlorantraniliprole and spinetoram were determined (Table 4). The results showed that the two resistant strains had no obvious cross-resistance to the three insecticides, with all cross-resistance ratios below 2.3, indicating minimal cross-resistance risk. Specifically, for the lufenuron-resistant strain (Resistance ratio = 2.89), the cross-resistance ratios to emamectin benzoate, chlorantraniliprole and spinetoram were 2.21, 1.67 and 1.90, respectively. For the chlorfenapyr-resistant strain (Resistance ratio = 3.40), the cross-resistance ratios to the three insecticides were 1.18, 1.37 and 1.48, respectively. These results indicated that the resistance mechanisms of *S. frugiperda* to lufenuron and chlorfenapyr are independent of the resistance mechanisms of the three reference insecticides. Therefore, the rotation and combined application of these insecticides in the field will not induce mutual resistance, which is conducive to resistance delay.

### 3.4. Biological Characteristics of the Lufenuron- and Chlorfenapyr-Resistant S. frugiperda Strains

The lufenuron-resistant strain adults laid significantly fewer eggs (*F*_2,87_ = 3.81, *p* = 0.03) and lower egg hatchability (*F*_2,87_ = 3.10, *p* = 0.05) than the susceptible strain, while the chlorfenapyr-resistant strain showed no obvious difference from the susceptible strain in these reproductive traits. For larval traits, the lufenuron-resistant strain had significantly shortened larval period (*F*_2,87_ = 4.00, *p* = 0.02) and reduced larval survival (*F*_2,87_ = 14.80, *p* < 0.001), whereas the chlorfenapyr-resistant strain did not differ significantly from the susceptible strain in these parameters (Table 5). Regarding pupation, the chlorfenapyr-resistant strain showed a significant decrease, while the lufenuron-resistant strain was comparable to the susceptible strain (*F*_2,87_ = 3.62, *p* = 0.03) (Table 5). Both resistant strains had significantly lighter pupal weight (*F*_2,87_ = 10.05, *p* < 0.001) (Table 5). The lufenuron-resistant strain had a significantly shorter pupal period, whereas the pupal duration of the chlorfenapyr-resistant strain was comparable to the susceptible strain. Adult lifespan of the lufenuron-resistant strain and chlorfenapyr-resistant strain was markedly shorter than the susceptible strain (*F*_2,87_ = 3.53, *p* = 0.03) (Table 5). No significant inter-strain differences were observed in adult emergence rate and sex ratio (Table 5). We also investigated the population survival dynamics and fitness metrics. Starting from identical newly hatched larvae, the lufenuron-resistant strain and chlorfenapyr-resistant strain exhibited consistently lower larval survival at all instars compared with the susceptible strain, with the lufenuron-resistant strain showing the lowest survival at each instar, followed by the chlorfenapyr-resistant strain, and both were significantly lower than the susceptible strain (1st instar: *F*_2,87_ = 37.33, *p* < 0.001; 2nd instar: 24.43, *p* < 0.001; 3rd instar: *F*_2,87_ = 89.02, *p* < 0.001; 4th instar:*F*_2,87_ = 104.97, *p* < 0.001; 5th instar: *F*_2,87_ = 104.32, *p* < 0.001; 6th instar: *F*_2,87_ = 94.20, *p* < 0.001) (Table 6). Consistent with the survival patterns, pupation counts of both resistant strains were significantly lower than that of the susceptible strain (*F*_2,87_ = 118.18, *p* < 0.001) (Table 6). The predicted next-generation larval quantity and net reproductive rate (R_0_) of resistant strains were far below the susceptible population. The relative fitness of the lufenuron-resistant strain and chlorfenapyr-resistant strain decreased significantly, demonstrating severe fitness trade-offs associated with resistance to lufenuron and chlorfenapyr (Table 6).

## 4. Discussion

In this study, toxicity bioassay was adopted to systematically evaluate the resistance profiles of four geographically distinct *S. frugiperda* field populations (collected from Jiangsu, Guangxi, Guizhou and Yunnan Provinces) against five commonly used insecticides, including lufenuron and chlorfenapyr. We further characterized the resistance development dynamics, cross-resistance risks and associated fitness costs of laboratory-selected resistant strains against lufenuron and chlorfenapyr. Integrating all experimental data, this work clarifies the low resistance evolution risk of Chinese field fall armyworm populations to the two target insecticides and provides actionable theoretical support for regional chemical control and resistance sustainable management. These findings are consistent with the general resistance and detoxification framework summarized by Kavallieratos et al., in which target-site mutations, metabolic detoxification, and fitness trade-offs jointly shape resistance evolution in *S. frugiperda* [5].

Consistent with global research reports, *S. frugiperda* populations across China have developed varying degrees of resistance to multiple chemical insecticides, with resistance levels presenting obvious regional heterogeneity driven by local cropping systems, pesticide application habits and environmental conditions [27]. Previous monitoring data indicated that fall armyworm populations in Fujian and Hunan Provinces generated medium-to-high resistance to organophosphorus insecticide acephate with resistance ratios exceeding 5-fold, while populations in Xinjiang and Gansu maintained relatively low resistance pressure despite a slow upward resistance trend [28]. In our field resistance survey, all tested field populations exhibited resistance ratios below 4 for lufenuron, chlorfenapyr, emamectin benzoate and spinetoram, remaining at susceptible or low-level insensitive status. Notably, Guangxi and Guizhou populations developed medium-level resistance to chlorantraniliprole with resistance ratios reaching 8.42 and 5.34, respectively, highlighting regional differential resistance pressure to diamide insecticides. Overall, lufenuron and chlorfenapyr still retain high field control efficacy against local fall armyworm populations in the four maize-producing provinces sampled in this research.

Lufenuron is a benzoylurea insect growth regulator that blocks chitin biosynthesis and disrupts normal molting and development of lepidopteran pests, which makes it a core chemical agent for fall armyworm management [29,30]. However, continuous single-agent application may gradually induce resistance risk in target pests [31,32]. In our 10 consecutive generations of indoor resistance selection, the resistance ratio of *S. frugiperda* to lufenuron only reached 2.89 after 10 rounds of screening, demonstrating extremely slow resistance accumulation and low short-term resistance evolution potential under sustained pesticide selection pressure. Chlorfenapyr acts as a mitochondrial uncoupler that disrupts pest energy metabolism; its unique action mode lacks single specific target proteins, which theoretically confers low resistance induction risk [33]. Previous overseas studies have recorded low-to-medium chlorfenapyr resistance in multiple mite and lepidopteran pests, yet our laboratory selection results showed that the resistance ratio of *S. frugiperda* to chlorfenapyr only rose to 3.40 after 10 generations of continuous screening, matching the low resistance risk conclusion drawn from Brazilian field monitoring of fall armyworm [34]. These combined results verify that lufenuron and chlorfenapyr possess inherent advantages for long-term rotational use in fall armyworm integrated pest management.

Rational rotation and mixture of insecticides with dissimilar modes of action represent the core strategy to delay pest resistance evolution, as resistance mechanisms targeting one insecticide class rarely confer adaptive advantages to agents with distinct action targets [35]. The three reference insecticides tested in this research cover three independent chemical families with unique action sites: emamectin benzoate belongs to avermectins targeting glutamate-gated chloride channels, chlorantraniliprole is a diamide insecticide acting on insect ryanodine receptors, and spinetoram targets nicotinic acetylcholine receptors [34,36,37]. Our cross-resistance determination revealed that cross-resistance ratios of the F10 lufenuron-resistant strain to the three insecticides were all lower than 2.3, and those of the chlorfenapyr-resistant strain remained below 2.0. The absence of significant cross-resistance indicates that the resistance genetic mechanisms of *S. frugiperda* to lufenuron and chlorfenapyr are completely independent of the adaptive pathways mediating tolerance to emamectin benzoate, chlorantraniliprole and spinetoram. Field rotation or combined application of these five insecticides will not accelerate mutual resistance development, which is beneficial for extending the service life of each chemical agent.

Resistance mutations frequently incur substantial fitness costs, defined as compromised survival, development and reproductive performance of resistant individuals in pesticide-free environments, which functions as a natural constraint limiting the rapid spread of resistant alleles in wild populations. The underlying mechanism mainly lies in excessive energy consumption of overexpressed detoxification enzymes and altered metabolic homeostasis caused by target-site mutations, which divert resources from growth and reproduction to pesticide detoxification [38]. Consistent with existing research on other insecticide-resistant *S. frugiperda* strains [39], our life-table comparison demonstrated comprehensive fitness disadvantages in both lufenuron- and chlorfenapyr-resistant strains. Resistant larvae exhibited lower survival rates, slower growth and lighter pupal weight; female adults showed reduced fecundity, shortened lifespan and decreased egg hatchability. Ultimately, the net reproductive rate of the lufenuron-resistant strain declined to 0.66 relative to the susceptible strain, and that of the chlorfenapyr-resistant strain dropped to 0.73. Such prominent fitness trade-offs greatly restrict the proliferation and dominant replacement of resistant individuals under natural field conditions without continuous pesticide selection, further lowering the overall resistance evolution risk of wild *S. frugiperda* populations to lufenuron and chlorfenapyr.

## 5. Conclusions

In summary, this study provides empirical evidence from major maize-producing regions in China confirming that *S. frugiperda* poses a low evolutionary risk of resistance to lufenuron and chlorfenapyr. While these data were generated in China, their implications extend well beyond national borders. Our findings offer a timely rationale for incorporating lufenuron and chlorfenapyr into early-stage rotation programmes in regions at an early stage of resistance development, before resistance alleles become established at high frequencies. Moreover, the significant fitness costs we quantified here provide critical baseline parameters for global researchers and pest managers to predict the dispersal dynamics of resistance alleles under field conditions across different agro-ecological zones. Taken together, the dual constraints of slow resistance accumulation and substantial fitness disadvantages position these two insecticides, with their distinct modes of action, as highly promising candidates for sustainable chemical control of *S. frugiperda* in the global pest management toolbox. Our study underscores that preserving such low-risk actives through globally coordinated rotation strategies—rather than relying on any single compound—is essential to prolong their effective lifespan and mitigate the ongoing crisis of insecticide resistance worldwide.

## Figures and Tables

**Figure 1:**
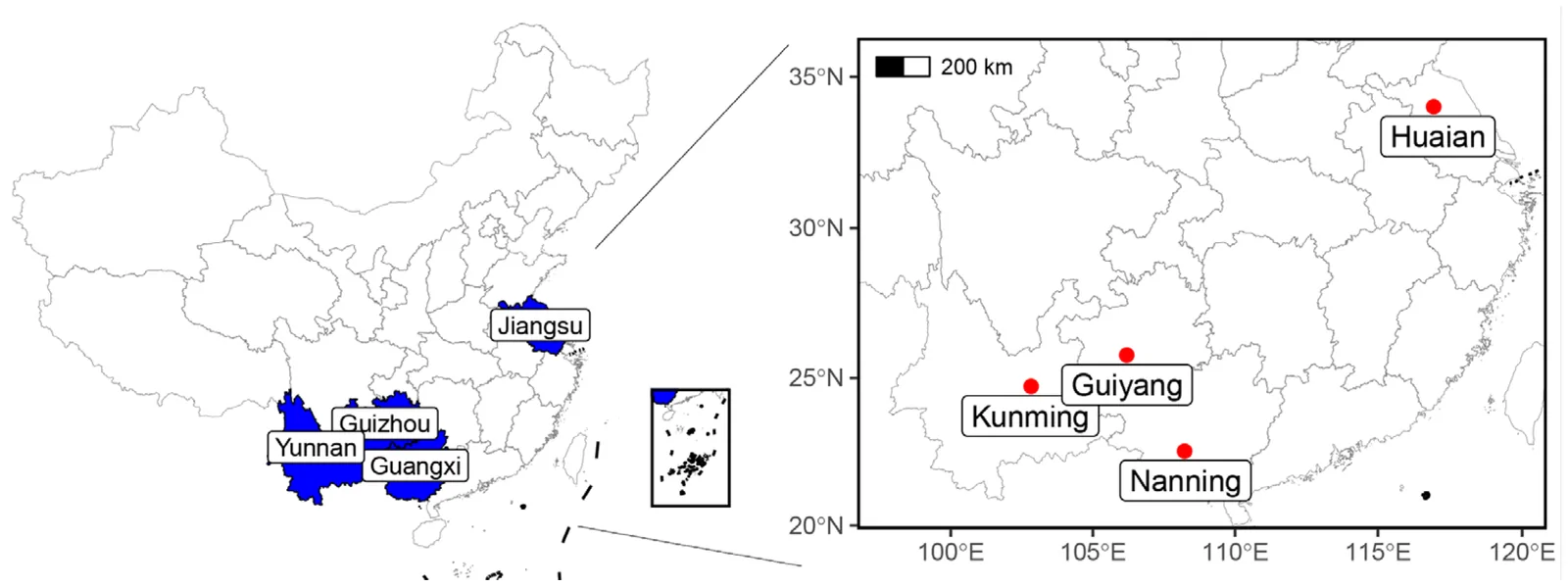
Map showing the collection sites of four field populations of *Spodoptera frugiperda* in China.

**Table 1:** Three-way ANOVA for the effects of region, insecticide, and concentration on corrected mortality of *Spodoptera frugiperda.*

Source of Variation	Sum of Squares	df	*F*	*p*
Region	0.171009	3	3.47	0.02
Insecticide	0.076461	4	1.16	0.33
Concentration	0.670790	1	40.81	<0.001
Region × Insecticide	0.409985	12	2.08	0.018
Region × Concentration	0.001674	3	0.03	0.99
Insecticide × Concentration	1.063247	4	16.17	<0.001
Region × Insecticide × Concentration	0.014324	12	0.07	1.00
Residuals	5.260052	320		

**Table 2:** Relative virulence of insecticides to different strains of *Spodoptera frugiperda.*

Insecticide	Population ^a^	LC_50_ (mg/L)	95% CL	Slope ± SE	RR ^b^
Lufenuron	Sus	45.83	29.58–71.02	1.20 ± 0.18	-
	JS	51.96	34.54–78.17	1.16 ± 0.17	1.13
	GX	129.98	97.86–172.65	0.99 ± 0.11	2.84
	GZ	92.99	69.89–123.74	0.99 ± 0.11	2.03
	YN	57.97	38.71–86.82	0.96 ± 0.15	1.26
Chlorfenapyr	Sus	5.84	3.82–8.94	0.83 ± 0.086	-
	JS	11.84	8.86–15.83	0.79 ± 0.063	2.03
	GX	21.48	16.91–27.29	0.76 ± 0.051	3.68
	GZ	6.17	4.19–9.075	0.86 ± 0.081	1.06
	YN	6.22	4.81–8.05	0.81 ± 0.051	1.07
Emamectin benzoate	Sus	3.47	2.37–5.10	0.87 ± 0.084	-
	JS	5.78	4.68–7.14	0.80 ± 0.044	1.67
	GX	8.32	6.53–10.59	0.71 ± 0.045	2.40
	GZ	5.43	4.60–6.41	0.84 ± 0.037	1.56
	YN	3.43	2.69–4.38	0.83 ± 0.051	0.99
Chlorantraniliprole	Sus	2.16	1.52–3.07	0.90 ± 0.073	-
	JS	1.65	0.89–3.052	0.93 ± 0.12	0.76
	GX	18.18	14.55–22.71	0.70 ± 0.036	8.42
	GZ	11.54	10.04–13.26	0.73 ± 0.026	5.34
	YN	1.54	0.82–2.88	0.92 ± 0.12	0.71
Spinetoram	Sus	0.21	0.17–0.26	0.74 ± 0.037	-
	JS	0.28	0.22–0.37	0.71 ± 0.044	1.33
	GX	0.76	0.52–1.13	0.61 ± 0.048	3.62
	GZ	0.43	0.32–0.59	0.62 ± 0.043	2.05
	YN	0.19	0.15–0.23	0.74 ± 0.038	0.90

Abbreviations: ^a^ Sus, susceptible strain; JS, Jiangsu province; GX, Guangxi province; GZ, Guizhou province; YN, Yunnan province; LC_50_, median lethal concentration; CL, confidence limits; SE, standard error; ^b^ RR, resistance ratio (=LC_50_ of field strain/LC_50_ of susceptible strain).

**Table 3:** Resistance screening of *Spodoptera frugiperda* larvae to lufenuron and chlorfenapyr.

Insecticides	Generation	LC_50_ (mg/L)	95% CL	Slope ± SE	RR ^a^
Lufenuron	F_0_	45.83	29.58–71.02	1.20 ± 0.18	-
	F_1_	50.04	33.24–75.34	1.26 ± 0.19	1.09
	F_2_	55.72	35.72–86.93	1.02 ± 0.17	1.22
	F_3_	66.57	44.75–99.03	1.01 ± 0.16	1.45
	F_4_	68.37	48.79–95.80	0.98 ± 0.13	1.49
	F_5_	74.35	53.59–103.14	1.02 ± 0.13	1.62
	F_6_	79.88	61.60–103.59	1.063 ± 0.11	1.74
	F_7_	91.53	70.25–119.25	1.20 ± 0.13	2.00
	F_8_	101.07	81.69–125.06	1.11 ± 0.095	2.21
	F_9_	110.59	88.96–137.49	1.16 ± 0.10	2.41
	F_10_	132.47	99.81–175.82	1.10 ± 0.12	2.89
Chlorfenapyr	F_0_	5.84	3.82–8.94	0.83 ± 0.086	-
	F_1_	6.29	4.45–8.88	0.78 ± 0.067	1.08
	F_2_	7.30	5.94–8.97	0.78 ± 0.041	1.25
	F_3_	8.10	6.24–10.51	0.79 ± 0.053	1.39
	F_4_	9.25	7.18–11.92	0.81 ± 0.055	1.58
	F_5_	10.22	7.70–13.55	0.82 ± 0.063	1.75
	F_6_	11.79	9.35–14.85	0.82 ± 0.052	2.02
	F_7_	13.21	10.38–16.82	0.82 ± 0.055	2.26
	F_8_	14.23	11.73–17.26	0.76 ± 0.041	2.44
	F_9_	16.71	14.01–19.93	0.80 ± 0.040	2.86
	F_10_	19.84	15.00–26.25	0.79 ± 0.063	3.40

Abbreviations: F_0_–F_10_, generations 0 to 10 of continuous selection; LC_50_, median lethal concentration; CL, confidence limits; SE, standard error; ^a^ RR, resistance ratio (=LC_50_ of Fn strain/LC_50_ of F_0_ strain).

**Table 4:** Cross-resistance to three insecticides in insecticide-resistant strains of *Spodoptera frugiperda.*

Insecticide	Population ^a^	LC_50_ (mg/L)	95% CL	Slope ± SE	RR ^b^
Emamectin benzoate	Sus	3.47	2.37–5.10	0.87 ± 0.084	-
	LR (F_10_)	4.08	3.06–5.45	0.78 ± 0.058	1.18
	CR (F_10_)	7.67	6.69–8.79	0.77 ± 0.028	2.21
Chlorantraniliprole	Sus	2.16	1.52–3.07	0.90 ± 0.073	-
	LR (F_10_)	2.95	2.34–3.70	0.76 ± 0.042	1.37
	CR (F_10_)	3.61	2.67–4.89	0.69 ± 0.053	1.67
Spinetoram	Sus	0.21	0.17–0.26	0.74 ± 0.037	-
	LR (F_10_)	0.31	0.24–0.39	0.60 ± 0.033	1.48
	CR (F_10_)	0.40	0.30–0.52	0.60 ± 0.036	1.90

Abbreviations: ^a^ Sus, susceptible strain; LR (F_10_), lufenuron-resistant strain after 10 generations of selection; CR (F_10_), chlorfenapyr-resistant strain after 10 generations of selection; LC_50_, median lethal concentration; CL, confidence limits; SE, standard error; ^b^ RR, resistance ratio (=LC_50_ of resistant strain/LC_50_ of susceptible strain).

**Table 5:** Comparison of biological characteristics between susceptible and resistant strains of *Spodoptera frugiperda* to lufenuron and chlorfenapyr.

Biological Characteristic	Susceptible Strain	Lufenuron-Resistant Strain (F_10_)	Chlorfenapyr-Resistant Strain (F_10_)
Egg production per female ± SE (eggs/head)	1207.25 ± 76.63 a	989.88 ± 45.61 b	1045.50 ± 42.01 ab
Egg period ± SE (days)	4.05 ± 0.16 a	3.55 ± 0.12 a	3.90 ± 0.18 a
Hatchability ± SE (%)	81.00 ± 1.05 a	76.13 ± 1.02 b	78.75 ± 1.88 ab
Larval period ± SE (days)	17.25 ± 0.31 a	15.63 ± 0.57 b	16.00 ± 0.33 ab
Larval survival rate ± SE (%)	84.25 ± 0.86 a	76.25 ± 1.23 b	79.75 ± 0.94 ab
Pupation rate ± SE (%)	82.00 ± 0.78 a	79.13 ± 0.81 a	79.25 ± 0.92 b
Pupal weight ± SE (mg)	244.00 ± 5.08 a	217.13 ± 3.85 b	225.00 ± 3.79 b
Pupal period ± SE (days)	11.50 ± 0.57 a	9.75 ± 0.45 b	10.50 ± 0.32 ab
Adult emergence rate ± SE (%)	83.75 ± 0.80 a	82.25 ± 0.41 a	82.75 ± 0.82 a
Sex ratio ± SE (female/male)	1.29 ± 0.01 a	1.24 ± 0.050 a	1.22 ± 0.05 a
Adult longevity ± SE (days)	17.88 ± 0.40 a	15.00 ± 0.46 b	15.50 ± 0.26 b

Abbreviations: Data are presented as mean ± standard error (SE). The susceptible strain is the laboratory unselected population maintained without insecticide exposure. The lufenuron-resistant strain (F_10_) and chlorfenapyr-resistant strain (F_10_) refer to the F_10_ generations derived from the susceptible strain after 10 consecutive generations of selection with the respective insecticide. Different lowercase letters within the same row indicate significant differences among strains (*p* < 0.05, Tukey’s HSD test).

**Table 6:** Life table of susceptible and resistant strains of *Spodoptera frugiperda* to lufenuron and chlorfenapyr.

Biological Characteristic	Susceptible Strain	Lufenuron-Resistant Strain (F_10_)	Chlorfenapyr-Resistant Strain (F_10_)
1st instar larvae count	100	100	100
2nd instar larvae survival	88.50 ± 0.43 a	82.50 ± 0.57 c	85.00 ± 0.45 b
3rd instar larvae survival	82.50 ± 0.57 a	75.83 ± 0.95 c	80.00 ± 0.86 b
4th instar larvae survival	79.50 ± 0.22 a	71.33 ± 0.71 c	76.17 ± 0.31 b
5th instar larvae survival	77.50 ± 0.43 a	67.00 ± 0.86 c	71.67 ± 0.33 b
6th instar larvae survival	76.00 ± 0.26 a	61.17 ± 1.30 c	66.67 ± 0.95 b
Pupation count	67.50 ± 0.62 a	55.50 ± 1.02 c	61.67 ± 0.42 b
Adult emergence count	62.50 ± 0.50 a	51.00 ± 0.58 c	53.67 ± 0.56 b
Predicted next generation larvae	42,468	27,896	30,836
Net reproductive rate (R_0_)	424.68	278.96	308.36
Relative fitness	1.00	0.66	0.73

Abbreviations: R_0_, net reproductive rate (=N_t+1_/N_t_, where N_t_ is initial number and N_t+1_ is the predicted next-generation larval number); relative fitness = R_0_ of resistant strain/R_0_ of susceptible strain. Different letters indicate significant differences among strains (*p* < 0.05, Tukey’s HSD test).

## Data Availability

Data are available from the corresponding author upon reasonable request.

## Abbreviations

The following abbreviations are used in this manuscript:

LC_50_	Lethal concentration
JS	Jiangsu
GX	Guangxi
GZ	Guizhou
YN	Yunnan
CL	Confidence limits
